# Participatory Inequality Across Countries: Contacting Public Officials Online and Offline

**DOI:** 10.1177/08944393211071067

**Published:** 2022-03-01

**Authors:** Shelley Boulianne

**Affiliations:** Department of Sociology, MacEwan University, Edmonton, AB, Canada

**Keywords:** contact officials, online political participation, political inequality, cross-national, socioeconomic status, age, gender

## Abstract

The Internet offers low-cost ways to participate in political life, which reduces the motivation required to participate and thus potentially reduces inequalities in participation. I examine online and offline contacting of elected officials using original survey data from Canada, France, the United Kingdom, and the United States collected in 2019 and 2021. Education is a consistent positive predictor of contacting in all countries as well as both modes of contact (online and offline). Income differences are small. Younger people are more likely to contact officials, online and offline, compared to older people. Females are less likely to contact officials, online and offline, compared to males. While political interest, efficacy, online information consumption, and online group ties are believed to lead to more equity in online communication, I do not see strong differences in these variables for online and offline contacting. I conclude by discussing the implications of exclusively online contacting of officials when this form of contact is devalued by elected officials, as well as the implications of participatory inequalities with respect to influencing public policy and access to government services.

## Introduction

Inequalities in political participation are important as they imply differential influence on public policy. In democratic systems, this inequality violates the principles of equality. Early discourse about the Internet offered optimistic accounts that inequality in political participation could be addressed by new, less resource-intense ways to participate in political life, such as contacting officials online. The lower effort was expected to diminish the observed inequalities in political participation that contribute to unequal voice and influence as well as unequal access to government services. Yet, this new online modality raises concerns about the differential impact of online versus offline communication in terms of influencing public policy.

This paper offers a systematic review of more than 20 studies exploring demographic differences in contacting officials online and offline. It demonstrates that online modes are more equitable than offline modes for younger people and those with less education, but the findings are less clear with respect to gender and income. Gender and income are as likely to matter in the online environment as in the offline environment; in other words, online contacting is not more equitable. This review uncovers a major gap in cross-national research about online contacting; to date, only single-country studies have been conducted on the topic of online contacting of government officials. This paper’s cross-national perspective enables us to understand whether the effects of digitization of citizen–government relations are consistently experienced across Western democracies or whether cross-national data show pre-existing patterns of socioeconomic differences in offline participation extend to the online realm.

I examine online and offline contacting using original survey data from Canada, France, the United Kingdom, and the United States collected in 2019 and 2021. In addition to replicating existing methodological approaches to resolve discrepant findings, I offer a distinct approach to inequalities by comparing patterns of offline contacting versus online contacting. This approach offers greater insight into the differential effects of demographic variables as well as key covariates (political interest, political efficacy, online news consumption, and online group ties) in the theory of digitalization of communication. Compared to offline contacting, online contacting is more equitable in terms of age and gender. These findings have implications for the state of democracy in these Western countries. Participatory inequalities lead to differential influence and access to government services; exclusively online contact may be less effectual than contacting offline or contacting through both modalities.

### Why Participation Inequality Matters

Democracy relies on the principle of equality (Dalton, 2017; Filetti, 2016). It requires that the preferences and interests of all citizens “be given equal consideration in the policy formation and the policy implementation process” (Schlozman et al., 2010, p. 488). Citizens ought to have an equal voice, as represented by one vote per person (Schlozman et al., 2018). The strength of a democracy depends on its ability to engage as many citizens as possible, which is the premise behind universal suffrage. Citizens’ participation makes for good government, as measured by the Economist Intelligence Unit (EIU) and World Bank Index (Dalton, 2017). Citizen engagement is linked to better policy outcomes, lower crime rates, prosperous communities, as well as healthier and happier citizens (Fishkin, 2009; Gastil et al., 2012; Putnam, 1993, 2000).

Some citizens are not engaged, which undermines democratic representation and can lead to biased policies. Those who participate will determine what social problems are addressed and exert influence on how these problems are addressed by the government (Dalton, 2017; Leighley & Oser, 2018; Schlozman et al., 2018; Verba et al., 1995). Research suggests that policy preferences differ for various socioeconomic groups (Dalton, 2017) and for those who participate versus those who do not (Leighley & Oser, 2018; Oser et al., 2014). As such, those groups that do not participate, for example, low-income earners and youth, have little influence on the government. Yet, these groups might require government assistance the most (Dalton, 2017; Schlozman et al., 2018); they may require government assistance to address episodes of unemployment or may require access to re-training or other educational opportunities to change their situation. In addition to contacting officials for these need-based reasons, youth and low-income citizens may contact officials to express their distinct views about the economic factors that caused their unemployment and/or their views about the role of government in offering re-training to support economic recovery and resilience.

### Contacting Officials (Online and Offline)

Contacting elected officials is an important activity for exercising voice and, thus, can speak to differential influence. In contrast to voting, contacting officials requires more skills and resources (Dalton, 2017). At the extreme levels, this activity could involve “prepar[ing] materials, identify[ing] relevant political actors, and mak[ing] a reasonable (and hopefully successful) presentation” (Dalton, 2017, p. 27). In contrast to voting, contacting officials offers more precise input into policy (Dalton, 2017; Schlozman et al., 2018) and, as such, the potential for influence on policy is much greater. Aars and Stromsnes (2007) suggest that contacting officials is a distinct form of influence, because it is “a kind of secret or invisible influence, and the contactors are not accountable to anyone but themselves” (p. 95). In other words, citizens can advance their own personal interests at the expense of the greater good. This activity is ideally suited to the comparison of online and offline inequalities because the activity easily translates across the modes, unlike voting that tends to be offline. It is also useful for cross-national comparisons of participation, in contrast to voting and donating to campaigns that have country-specific rules.

Verba et al. (1995) explain citizen’s participation in terms of time, money, and skills and the lack of participation in terms of “they can’t, they don’t want to, and nobody asked” (p. 16). The Internet lowers the effort required to participate and offers a convenient mode to contact public officials and make campaign donations (Schlozman et al., 2010, 2018). As such, the strong role of political interest on participation (Smets & van Ham, 2013) may be smaller in online modalities. The Internet can provide easy access to a wealth of information, which also increases political interest and, thus, motivation to participate (Beam et al., 2018; Boulianne, 2011; Schlozman et al., 2010, 2018). Access to online information contributes to resources supporting participation. Last, the Internet provides another forum for political parties and organizations to recruit citizens to participate—addressing the “nobody asked” explanation (Best et al., 2008; Bimber, 1999; Gibson et al., 2005; Park & Perry, 2008; Schlozman et al., 2010, 2018; Verba et al., 1995). In this context, civic and political groups can encourage their members to contact elected officials to influence decisions; these groups can pool resources, cultivate skills, and help mobilize the participation of people who would not do so otherwise (Boulding & Holzner, 2020).

Within this body of research is an ongoing debate about whether online modes are as impactful on the direction of government compared to offline modes. Buchi and Vogler (2017, p. 3) argue that “since direct influence on policy and government is primarily achieved through offline modes, online participation is more effective when it translates to traditional offline participation.” Some scholars have found that politicians are dismissive of online forms of contact (Chen et al., 2018). As such, if a particular group, such as youth, is only contacting officials online and not offline, their voice may not be heard as strongly as those who use offline forms or engage in both online and offline forms of contacting. Studies about the perceived influence of online forms of participation do not show age differences, but do show gender differences (Demertzis et al., 2013; Lee & Huang, 2014; Martin et al., 2018); but, notably, none of these studies were completed in Western democracies. The implication of relying exclusively on online contacting is that the degree of influence on public officials may be diminished. A large body of research has already established that youth opt for online political activities more so than other age groups (Boulianne & Theocharis, 2020). As such, while online contacting may be easier and preferred by this group, their policy preferences, such as concern about the environment (Franzen & Meyer, 2010), may not be considered by public officials.

RQ1: Do political interest, efficacy, online news use, and online group ties differ in their correlation with online contacting as opposed to offline contacting?

### Demographic Differences

Despite statistical models that account for motivation, skills, and resources, participatory inequality persists in relation to demographic differences. Optimists hoped that the Internet would be a “circuit breaker,” disrupting the effects of socioeconomic status (SES) on political activity (Schlozman et al., 2010, p. 488). Early work highlighted the Internet’s potential to “redistribute political power, break the monopolistic positions of traditional elites and media, and amplify the voice of the common citizen” (Buchi & Vogler, 2017, p. 2). One of the major impediments to this potential has been the unequal distribution of Internet access (Mossberger et al., 2008) and online skills (Beam et al., 2018; Buchi & Vogler, 2017; Dalton, 2017; Hargittai, 2002, 2010). Inequalities in online access and digital skills map onto existing structures of social inequality.

People are more likely to participate in politics if they have resources, such as time, money, and civic skills (Dalton, 2017; Nie et al., 1996; Putnam, 2000; Schlozman et al., 2010, 2018; Verba et al., 1995). Resource theories suggest the likelihood of engaging in politics should increase with levels of education and income. Education matters for political participation for several reasons. Completion of higher education develops civic skills and knowledge that relate to participation in political activities (Buchi & Vogler, 2017; Dalton, 2017; Nie et al., 1996). Higher SES can also enhance one’s understanding of how the government works, which can help with “navigating the complex political world-knowing how to lobby the city council, contact their member of parliament, or even write a blog on political topics” (Dalton, 2017, p. 57). Education also helps develop networks that increase the chance of being asked to participate in politics, including collective action (Nie et al., 1996; Olcese et al., 2014). Finally, college attendance may help cultivate a sense of civic duty to participate (Youniss, 2009).

Schlozman et al. (2010, 2018) explore the stratification of online and offline political activities by SES (and age), using 2008 and 2012 Pew data. They document the persistence of socioeconomic differences in online and offline political participation and demonstrate that the digital divide further diminishes opportunities for equal participation. They conclude that “although these revolutionary technological changes provide citizens with new means of expressing political voice, they have not severed the deep roots that anchor political participation in social class” (Schlozman et al., 2018, p. 128).

Offering a more international perspective on this topic, Dalton (2017) documents the role of SES in predicting a wide range of offline political activities. The conclusion replicates Schlozman et al.’s (2018) findings: there is a participation gap in offline activities defined by SES. However, relying on International Social Survey Programme (ISSP) and other international data sources limits the analysis of differences in online participation because these international surveys have few measures of online political activities (Boulianne, 2020). The present cross-national perspective enables us to understand whether the effects of digitization of citizen–government relations are experienced consistently across Western democracies. Theories related to motivations, skills, and resources would suggest these effects should be consistent across countries.

To offer a more comprehensive picture about participatory inequality across online and offline activities, I aggregate the findings across the studies that have looked at both online and offline forms of contacting (see Table 1). I look at these findings in aggregate, summarizing the tendencies to report significant differences. The meta-data suggest consistent patterns of inequality defined by education. This finding is consistent across a variety of countries tested (see Table 1). For offline forms of contacting, 17 of 21 studies (81%) find education has a significant impact. For online forms of contacting, six of nine studies (67%) suggest education has a significant impact.

**Table 1.** Literature Summary of Contacting Officials.

		Education		Income		Age		Gender: Female = 1	
Reference	Country, data source	Online	Offline	Online	Offline	Online	Offline	Online	Offline
Aars & Stromsnes, 2007	Norway 2001		*Ns*		Yes		*Ns*		*Ns*
Anduiza et al., 2010	Spain 2007	Yes		Yes		Yes		*Ns*	
Best et al., 2008	USA 2007	Yes		*Ns*		*Ns*		*Ns*	
Bimber, 1999	USA 1996–98	*Ns*	*Ns*			Yes	Yes	Yes	*Ns*
Boulding and Holzner, 2020	LAPOP 2006–14		Yes		Yes		Yes		*Ns*
Brundidge et al., 2013	USA 2008	Yes	Yes	Yes	Yes	Yes	Yes	*Ns*	Yes
Cao and Brewer, 2008	USA Pew 2004		Yes		*Ns*		Yes		*Ns*
Carreras, 2018	LAPOP 2004–14		Yes		Yes		Yes		*Ns*
Carreras, 2018	ESS 2002–14		Yes		Yes		Yes		Yes
Carreras, 2018	ISSP 2014		Yes				Yes		Yes
Dalton, 2017	USA Pew 2008	Yes (.21)	Yes (.14)	Yes (.15)	Yes (.07)	*Ns (.04)*	Yes (.12)	*Ns (-.03)*	*Ns (-.01)*
Dalton, 2017	USA Pew 2012	Yes (.24)	Yes (.18)	Yes (.06)	*Ns (.02)*	*Ns (.05)*	Yes (.08)	*Ns (-.02)*	*Ns (-.01)*
Gibson et al., 2005	UK 2002	*Ns*	*Ns*			*Ns*	Yes	Yes	*Ns*
Grasso, 2016	ESS 2002–06		Yes				Yes		Yes
Ha et al., 2013	S. Korea		*Ns*		*Ns*		Yes		*Ns*
Hsieh & Li, 2014	Taiwan 2008	Yes		*Ns*		Yes		Yes	
Huang et al., 2017	Asia Barometer 2010–12		Yes		*Ns*		Yes		Yes
Li & Marsh, 2008	UK 2001		Yes				Yes		Yes
Lockwood & Krönke, 2021	Afrobarometer 2014–15		Yes		*Ns*		Yes		Yes
Mattes, 2015	South Africa 2011		Yes						*Ns*
Mattila & Papageorgiou, 2017	ESS 2002–12		Yes				Yes		Yes
Park & Perry, 2008	ANES 1996–2004		Yes		*Ns*		*Ns*		*Ns*
Stockemer & Rapp, 2019	ESS 2014		Yes				Yes		Yes
Sylvester & McGlynn, 2010	USA Pew 2007			*Ns*	*Ns*	Yes	Yes	*Ns*	*Ns*
Thananithichot, 2012	Thailand 2011		Yes		*Ns*		*Ns*		Yes
Vaccari et al., 2015	Italy 2013	*Ns*		*Ns*		Yes		Yes	
Summary		6 of 9 studies “yes”	17 of 21 studies “yes”	4 of 8 studies “yes”	6 of 14 studies “yes”	6 of 10 studies “yes”	18 of 21 studies “yes”	4 of 10 studies “yes”	10 of 22 studies “yes”
		67%	81%	50%	43%	60%	86%	40%	45%

Ns refers to not significance at the .05 level. Dalton (2017) analyzes the ISSP data, which are also analyzed by Carreras (2018); to avoid duplication of data sources, I only itemize the results from Carreras. Also, Dalton (2017) reports standardized coefficients and does not report tests of significance. As such, the authors have made decisions about whether the standardized effect would reach the .05 threshold for significance. The summary is limited to studies using multivariate analysis (at least two demographic variables) of contacting officials. As such, Schlozman et al.’s (2018) bivariate analysis of Pew and American National Election Study (ANES) data is excluded from the above summary. The studies were compiled by consulting systematic reviews and meta-analysis in this field of research (Boulianne, 2020; Rueß et al., 2019) and a Scopus search on May 4, 2021 using the search terms: contacting AND (government OR officials OR politicians OR representatives) in the social sciences database. Furthermore, I focused on studies published since 1999 when the study of online versus offline contacting began with Bimber (1999). I excluded studies based on students because these studies could not provide estimates on education, income, and age effects, given the homogeneity within this subset of the population.

This meta-view suggests educational differences are less frequently significant in the online environment compared to the offline environment. As such, the first hypothesis builds from a well-established body of research about socioeconomic differences, but extends this research into the online realm and provides a cross-national perspective. Based on the theory about online contacting requiring lower resources and effort as well as existing meta-data, the first hypothesis is:

H1: Education is a weaker predictor of online contacting of officials compared to offline contacting in four Western democracies.

As for income, the meta-data suggest about half of studies find significant income differences in contacting online or offline. As mentioned, access to government officials is important for lower-income people who may need assistance due to unemployment or re-training. For offline contacting, six of 14 studies (43%) find significant income differences, whereas for online contacting four of eight (50%) studies find significant differences. These findings do not offer strong support for the differential impact of income in the online versus offline context. In addition, the effects of income are not clear across different countries. As mentioned, this finding has distinct implications on public policy and access to government services. Low-income people may be in a distinct position of high need for government assistance as well as hold distinct policy views about the economy and education systems. Continuing the theory that online forms require lower resources, I hypothesize that income differences are weaker in the online environment compared to the offline environment.

H2: Income is a weaker predictor of online contacting of officials compared to offline contacting in four Western democracies.

The meta-data also point to the importance of age in predicting online and offline forms of contacting officials. Key life events would provide incentives to contact the government for assistance as well as to influence public policy, including the need for social assistance related to retirement or old age. In addition to accessing these resources, older people often form associations or lobby groups to formally influence government, as illustrated by the US-based American Association of Retired Persons (AARP). The existing research suggests that, in the offline context, age is a consistent predictor of contacting officials. Of the 21 studies testing age effects, 18 (86%) found age predicts offline contacting.

In terms of online contact, six of 10 studies (60%) found age predicted online contacting. As observed with education, online forms of participation seem to be more equitable compared to offline forms based on an analysis of existing research. As mentioned, younger adults are distinctive in their preference for the online modality of participation and in their skillful use of technology (Boulianne & Theocharis, 2020). Younger generations learn new technologies in schools, making them comfortable using digital media outside of school and for a variety of purposes. This greater skill and comfort with technology may increase their likelihood of participating in online political activities compared to offline activities (Boulianne & Theocharis, 2020). Beam et al. (2018) find that digital skills can make up for a lack of education; those with greater digital skills are more likely to participate online, even if they have lower education. Historically, youth have been less likely to contact officials and participate in politics (more generally) due to a lack of political interest and knowledge (Holt et al., 2013). As such, the diminished inequalities in online contacting offer some fuel for optimists, but also, as mentioned, concerns exist regarding whether online contacting efforts are as influential on government officials. The next hypothesis is:

H3: Age is a weaker predictor of online contacting of officials compared to offline contacting in four Western democracies.

Gender differences in political participation are, in part, explained by gender differences in income (Brundidge et al., 2013). However, gender differences also relate to gendered experiences in socialization, which result in women being less interested, less efficacious, and less knowledgeable about politics (Carreras, 2018). If the Internet lowers the level of effort to participate and thus reduces the motivation to participate, this online modality should see a smaller gender gap in participation. Brundidge et al. (2013) find gender differences in offline, but not online, contacting of officials in the United States. However, other scholars find the reverse (Table 1). Similar to income, the literature does not offer strong support for the differential impact of gender in the online versus offline context.

Gender differences might relate to the perceived efficacy of online activities (Carreras, 2018; Heger & Hoffmann, 2019), as gender differences in the perceived ability to influence government may result in differences in contacting. As mentioned in relation to youth and online contacting, many question the impact of online activities in contrast to their offline manifestation (Buchi & Vogler, 2017). Many studies show women are less likely to think the Internet improves citizens’ influence on government (Demertzis et al., 2013; Lee & Huang, 2014; Martin et al., 2018). In this case, women may be less motivated to participate in online contacting compared to offline contacting. Early research about gender and political participation found that gender differences disappeared once political efficacy, political interest, and information were added to the model (Verba et al., 1997). Among the studies summarized in Table 1, nine consider political efficacy; gender differences remain statistically significant in six of these nine studies, suggesting political efficacy is not the primary explanation for gender differences. The final hypothesis is:

H4: Gender is a weaker predictor of online contacting of officials compared to offline contacting in four Western democracies.

### Cross-National Differences

Table 1 summarizes seven studies using cross-national data, but none examine both online and offline contacting. The cross-national comparative approach can help to identify whether the patterns of participatory inequality are country-specific (and thus require theorizing about political context) or consistent across countries, which would provide insights into a theory about technological affordances (lower resources, easier access, digital skills). These studies offer some insights into cross-national differences, but not in terms of the importance of age, gender, education, and income differences in contacting online versus offline.

In terms of participation inequalities, we expect variations in these countries because existing research has found the participation gap is linked to the strength of the welfare system (Dalton, 2017). Reducing social inequality by redistributing wealth and ensuring access to quality, publicly funded education is expected to reduce political inequality in terms of who participates. Compared to Canada, the United Kingdom, and France, the United States has a weak welfare system and, as such, we might expect more political inequality to match greater socioeconomic inequality.

A handful of studies examine online and offline contacting; these studies are exclusively conducted in the United States (Table 1). As such, we know about patterns of differences in online and offline contacting in the United States, but not in other countries. A cross-national study could help test whether the theory about digital political participation applies across countries, supporting theories of technological affordances. However, country-specific patterns of participatory inequality would suggest online transformations to participation will depend on political context.

Cross-national differences for age and gender are less clear. For gender and online contacting, differences were observed in the United States, the United Kingdom, Taiwan, and Italy, but not Spain (Table 1). We do not know if something is different about the structural opportunities for online contacting in Spain compared to these other countries. The studies offer different results; this is a conclusion of all systematic reviews and the meta-analysis. Sometimes the deviant cases are statistical anomalies and other times are valuable evidence to support more nuanced theories. For age and online contacting, differences were observed in the United States, Spain, Taiwan, and Italy, but not the United Kingdom (Table 1). Again, we do not know if something is different about the structural opportunities for online contacting in the United Kingdom compared to these other countries that helps explain this lack of age difference. This finding could be an anomaly. As mentioned, no studies use cross-national data to test online contacting. Testing these relationships within multiple Western democracies will provide insights about whether the theory about online participation being less resource-intense, easier, and, thus, more equitable can be tested. As such, I offer a final research question:

RQ2: Are there cross-national differences in predictors of online contacting?

## Methods

### Sample

Lightspeed Kantar Group administered a survey to an online panel in September to November 2019 and again in February 2021 to a new group of respondents. I pooled the sample into one large database, and then included the year of data collection as a statistical control. In total, 12,359 respondents are included in the analysis. Quotas were in place to ensure the online panel matched census data for each country (see Table 2). The sample sizes are similar across the four countries: Canada (*n* = 3107), France (*n* = 3010), the United Kingdom (*n* = 3042), and the United States (*n* = 3200). The survey was conducted in English and French. Lightspeed Kantar uses a weighting efficiency, rather than response rate, to report on sample quality. This metric measures the match between the census profile and sample characteristics. The weighting efficiencies were between 97% and 99% for each country and each year. Due to the close match between the census profiles and sample characteristics (see Table 2), I did not weigh the data.

**Table 2.** Descriptive Statistics.

		USA official (%)	USA survey	UK official (%)	UK survey	France official (%)	France survey	Canada official (%)	Canada survey
Gender	Male (0)	**49**	48%	49	51%	**48**	49%	**49**	47%
	Female (1)	**51**	52%	51	49%	**52**	51%	**51**	53%
Age groups	18–24	**12**	12%	**11**	11%	**10**	10%	**11**	10%
	25–34	**18**	18%	**17**	17%	**15**	15%	**16**	17%
	35–44	**16**	16%	**16**	16%	**16**	16%	**16**	16%
	45–54	**16**	17%	**18**	18%	**17**	17%	**18**	18%
	55–64	**17**	14%	**15**	17%	**16**	19%	**17**	16%
	65 years+	**21**	24%	**22**	21%	**25**	23%	**21**	23%
Age6gr (0.5)	Average (SD)		2.75 (1.72)		2.75 (1.67)		2.88 (1.66)		2.84 (1.66)
Income quintiles (0–4)	Average (SD)		2.02 (1.39)		2.00 (1.43)		1.94 (1.31)		2.05 (1.37)
Education (1–4)	Average (SD)		2.13 (1.10)		1.86 (1.05)		1.78 (1.02)		1.96 (0.98)
Student (0.1)			4%		4%		6%		6%
Unemployed (0.1)			14%		11%		7%		8%
Retired (0.1)			29%		25%		30%		30%
Political interest (1–4)	Average (SD)		2.76 (0.99)		2.57 (0.93)		2.35 (0.97)		2.57 (0.93)
Political efficacy (1–4)	Average (SD)		2.43 (0.90)		2.16 (0.82)		1.96 (0.84)		2.34 (0.84)
Time using online news (1–8)	Average (SD)		3.12 (1.83)		2.84 (1.49)		2.93 (1.51)		2.94 (1.60)
Following civic groups on social media (0.1)			40%		39%		38%		41%
Contact offline (0.1)			28%		20%		21%		20%
Contact online (0.1)			35%		28%		26%		25%

Official statistics determined from the following sources: US Census (2019), Office of National Statistics (2016), National Institute of Statistics and Economic Studies (2018), and Statistics Canada (2016).

### Education

For education, I asked about the highest level of education completed. I provided country-specific categories, but recoded these into four categories: high school diploma or less, some college, bachelor’s degree, and more than a bachelor’s degree. For the pooled sample, the average is 1.94 and standard deviation is 1.05 (*n* = 12,359).

### Income

For income, I asked about the household’s combined yearly income before taxes, including employment, pensions, investments, or any other source. I created quintiles from this distribution with approximately 20% of the sample in each quintile for each country. The quintile approach is appropriate given that each country uses a different currency. Quintiles are used in the studies cited in the literature review (Boulding & Holzner, 2020; Schlozman et al., 2010, 2018). The country-specific averages are listed in Table 2; for the pooled sample, the average is 2.00 and standard deviation is 1.37 (*n* = 11,493).

### Gender

Across the four countries, the sample includes similar portions of males and females. I also offered a non-binary category but only 55 of 12,359 respondents chose this response; given the small number, I could not include this gender category into the regression model. For the pooled sample, 51% self-identified as female.

### Age

For age, I asked people’s birth year. I used these responses to create six age groups. For the pooled sample, 11% are 18–24 years, 17% are 25–34 years, 16% are 35–44 years, 17% are 45–54 years, 16% are 55–64 years, and 23% are 65 years or more.

### Student, Retirement, and Unemployment Status

While the research questions and hypotheses focus on the above demographics, I introduce a series of statistical controls to help isolate the effects of these variables from other correlated processes, including life events (retirement, pursuing advanced education, and unemployment). For retirement, student status, and unemployment status, I asked, “Please select the one that applies to how you spend the majority of your time.” For this question, people who are students are coded as 1 (others as zero), unemployed coded as 1 (others as zero), and retired coded as 1 (others as zero). In the pooled sample, 5% are students, 10% are unemployed, and 29% are retired.

### Political Interest

For political interest, I asked, “How interested would you say you are in politics?” Respondents could choose: not at all interested, not very interested, fairly interested, and very interested. On this four-point scale, the average level of political interest is 2.57 (SD = .97). Studies consistently find political interest correlates with contacting officials, but demographic differences persist despite the inclusion of political interest (Aars & Stromsnes, 2007; Best et al., 2008; Boulding & Holzner, 2020; Cao & Brewer, 2008; Lockwood & Krönke, 2021; Mattila & Papageorgiou, 2017; Park & Perry, 2008; Thananithichot, 2012).

### Online News

For online news consumption, I asked about time spent (in a typical day) using the Internet for news about political and current affairs. Respondents were offered a series of response options including never, less than 30 minutes, 30 minutes to 1 hour, 1–1.5 hours, 1.5–2 hours, 2–3 hours, 3–4 hours, and more than 4 hours. The average was 2.96 (SD = 1.61), which corresponds to 30 minutes to 1 hour. Several studies examine online news as a measure of resources facilitating the contacting of officials, with some studies finding significant correlations (Hsieh & Li, 2014; Park & Perry, 2008) and others not (Cao & Brewer, 2008; Vaccari et al., 2015).

### Political Efficacy

For political efficacy, I presented the statement, “People like me can influence government.” Respondents could choose to respond as strongly disagree, disagree, agree, and strongly agree. On this four-point scale, the average level of political efficacy is 2.23 (SD = 0.87). Political efficacy is also correlated with contacting officials. Again, studies account for the role of political efficacy, but demographic differences persist (Aars & Stromsnes, 2007; Dalton, 2017; Li & Marsh, 2008; Lockwood & Krönke, 2021; Mattila & Papageorgiou, 2017; Thananithichot, 2012). Three studies found political efficacy was not a significant predictor of contacting officials (Hsieh & Li, 2014; Park & Perry, 2008; Vaccari et al., 2015).

### Following Groups on Social Media

Several studies consider organizational memberships (Aars & Stromsnes, 2007; Boulding & Holzner, 2020; Gibson et al., 2005; Huang et al., 2017; Thananithichot, 2012) and tend to find significant effects on patterns of contacting. For organizational ties, I offer a distinct perspective moving beyond the membership-based organizational focus of existing literature (Best et al., 2008; Bimber, 1999; Gibson et al., 2005; Park & Perry, 2008; Schlozman et al., 2010, 2018; Verba et al., 1995). Instead, I asked whether people followed various groups (leisure groups, such as a cultural group, hobby group, or sports group; environmental groups; charity groups; and groups that help or rescue animals) on social media. The number of groups were counted, but the resulting distribution was highly skewed, so the variable was dichotomized into whether they follow any groups (0 = no groups, 1 = any civic or social group). Approximately 40% of respondents followed a group on social media. Table 2 contains the country-specific results for this variable.

### Contacting Officials

For contacting officials, I asked about online (by email, social media) and offline (by letter or telephone) forms of contacting elected officials during the past 12 months. The response options were never, rarely, from time to time, and often. However, given the extreme skew in this distribution, I did not present ordinal logistic regression, but instead opted for binary logistic regression. For the pooled sample, 28.42% had contacted online and 22.45% had contacted offline. Table 2 presents the descriptive statistics related to contacting officials in each country.

### Analysis

**Figure 1.**
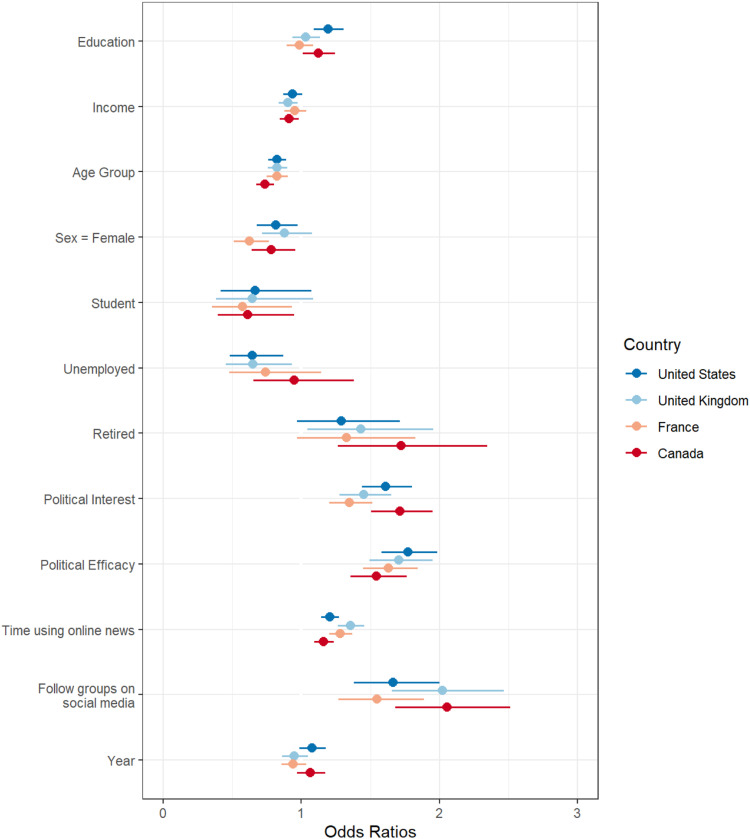
Cross-national comparsion of online contact.

**Figure 2.**
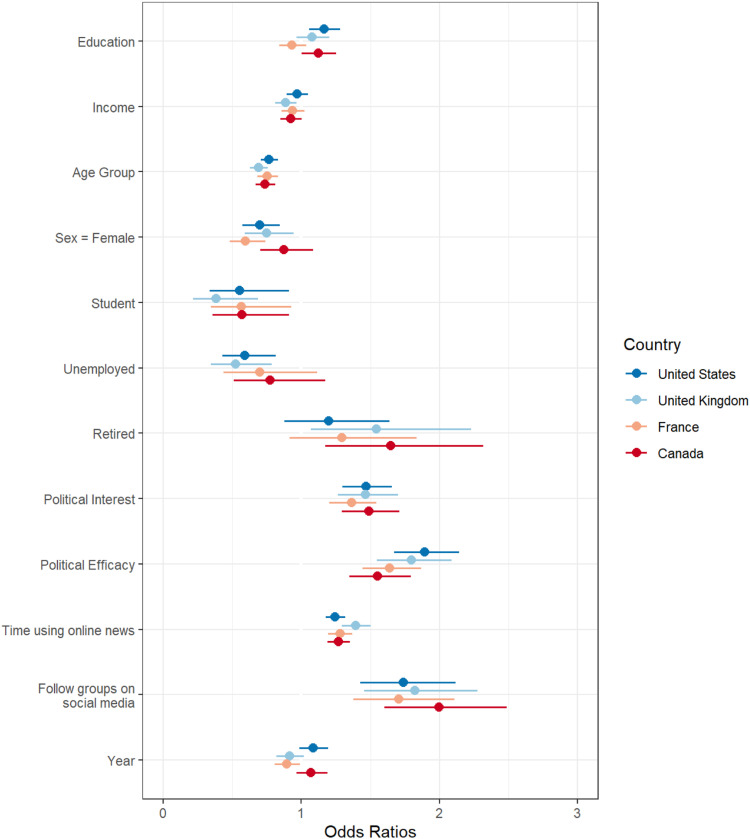
Cross-national comparsion of offline contact.

**Figure 3.**
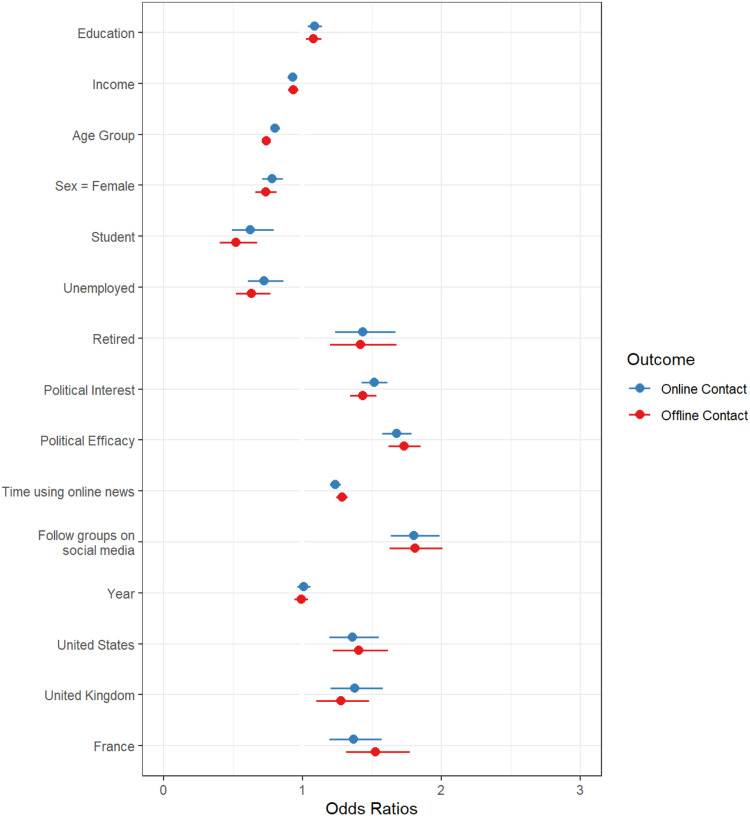
Comparsion of online and offline contact model coefficients.

The analysis proceeds in a series of steps. First, logistical regression models are produced that estimate the role of demographic variables and other variables on contacting. The analysis separates each country and online versus offline contacting. This approach replicates existing methodological approaches comparing online and offline contacting in single countries (Bimber, 1999; Brundidge et al., 2013; Dalton, 2017; Gibson et al., 2005; Sylvester & McGlynn, 2010). These results are illustrated in Figures 1 and 2. However, this approach is not ideal for comparing the effects of demographic variables on online versus offline contacting, which is the core focus of the research questions and hypotheses. As such, Figure 3 includes a logistic regression analysis with pooled results across countries; the figure compares the coefficients for online versus offline contacting. As a final test, these pooled results are used in a multinomial logistic regression with offline (only) contacting as the reference group. This analysis offers a direct comparison of the effects of demographic variables on online versus offline contacting. Table 3 compares offline (only) to online contacting (only) using multinomial logistic regression.

**Table 3.** Multinomial Logistic Regression of Online Versus Offline Contacting.

	Online only (8.1%) with Offline only (2.0%) as Reference Group			
	b	SE	ExpB	p
Education	0.033	0.079	1.034	.673
Income quint	0.036	0.063	1.037	.569
Age6gr	0.279	0.069	1.322	<.001
Females1	0.416	0.163	1.515	.011
Student1	1.013	0.470	2.753	.031
Unemployed1	0.308	0.277	1.361	.266
Retired1	−0.207	0.254	0.813	.414
United States	−0.131	0.217	0.877	.546
United Kingdom	0.243	0.232	1.275	.294
France	−0.243	0.227	0.784	.284
Political interest	0.150	0.097	1.162	.121
Political efficacy	0.191	0.099	1.211	.054
Time using online news	−0.109	0.048	0.897	.024
Civic groups on SM	−0.101	0.162	0.904	.533
Year 2021	−0.028	0.077	0.973	.722

SM, social media. Full model is available in Appendix D. Canada is the reference group for the above model.

### Ethics

This research was reviewed and approved for ethical considerations by MacEwan University (101662 and 101856) in accordance with Canada’s *Tri-Council Policy Statement: Ethical Conduct for Research Involving Humans (TCPS)*. Data and replication files are available: https://doi.org/10.6084/m9.figshare.19093499.v1

## Results

The first hypothesis is about education being a weaker predictor of online contacting of officials compared to offline participation in four Western democracies. For the United States and Canada, the hypothesis is not supported; educational differences are similar in magnitude in the online environment compared to the offline environment (Figures 1 and 2; Appendix A). More-educated Canadian and Americans are more likely than less-educated Canadian and Americans to contact officials online and offline. In the United Kingdom and France, the hypothesis is also not supported. In these countries, educational differences are not significant in the online or offline environment. In sum, Hypothesis 1 is not supported; educational differences are not significant in the United Kingdom or France, and educated people are as likely to contact officials online as offline in the United States and Canada.

For income differences, I expect the role of income to be smaller in the online context compared to the offline context. In France and the United States, income is not a significant predictor of offline or online contacting of officials. Income is a significant predictor in the United Kingdom and Canada, but the magnitude of the effect is comparable in online and offline environments. Furthermore, the patterns suggest people with higher income are less likely than people with lower income to contact officials. Hypothesis 2 is not supported.

Hypothesis 3 is about age being a weaker predictor of online contacting of officials compared to offline contacting in four Western democracies. Age is a consistent predictor in all models and in all countries. Younger people are more likely to contact officials in all models. The age differences are smaller in the online context compared to the offline context in all countries except Canada (where the effect size is the same).

Hypothesis 4 is about gender and contacting officials. This hypothesis finds support in the United States and United Kingdom, where gender differences are smaller in the online mode compared to the offline mode. In France, the gender gap is equivalent in both modalities. In Canada, offline contacting is more equitable for males and females than the online environment (Figures 1 and 2; Appendix A). In Canada, the United States, and France, females are less likely to contact officials online than males. In sum, in relation to Research Question 2 about cross-national differences, gender seems to differ in its effects more than other variables in the different countries.

The model fit is best in the United States (r-square of .223 for the online model and .232 for the offline model). In all countries, political interest, political efficacy, online news, and following civic groups on social media positively relate to the likelihood of contacting officials (RQ1). These variables are important to the overall model fit, but for the most part do not explain away demographic differences. Age and gender differences persist (H3 and H4). These variables operate differently in the different countries (RQ1 and RQ2). For example, political interest is a weak (but still positive and significant) predictor of online contacting in France, but the relationship is strongest in Canada. However, overall the estimated effects of these sets of variables are within the 95% confidence intervals, suggesting small differences.

Figures 1 and 2 replicate existing modeling approaches, allowing a clear connection between these new results and existing scholarship (Table 1) and adding a much-needed cross-national comparative perspective about inequality in online and offline contacting. However, this approach does not isolate the distinctiveness of online contacting as separate from offline contacting.

In Figure 3, I pooled the results across countries and present a side-by-side comparison of online and offline contacting of officials (see Appendix B). Pooling the sample helps to reveal the big picture with respect to the four key hypotheses. In these models, countries are added as statistical controls. Canada is the reference group and the other countries are converted into dummy variables. Compared to other countries, Canadians are less likely to contact officials online and offline. This presentation clearly illustrates that education and income have comparable roles in online and offline contacting. Age and gender, on the other hand, have different roles in online and offline contacting (H3 and H4). In terms of political interest, efficacy, and following groups on social media, the results are quite similar (RQ1). Online news consumption has a slightly stronger role in offline contacting compared to online contacting.

As mentioned, other statistical controls were included in the models to account for life events that may impact the likelihood of contacting officials (Figure 3, Appendix B). Being a student reduced the likelihood of contacting officials. The finding is surprising because being a student might increase the skills and political interest to contact officials, similar to education; however, the two variables work in different directions. Education has a positive effect on contacting officials, whereas being a student has a negative effect. Unemployment and income are also linked, but the findings are in different directions. Lower-income people are more likely to contact officials, but those who are unemployed are less likely to contact officials. Being retired, which is a variable linked to age, increases the likelihood of contacting officials, despite age coefficients suggesting older people are less likely than younger people to contact officials. While the COVID-19 pandemic moved many activities online that were previously offline, I did not find significant changes in online contacting between 2019 and 2021. Appendices C and D include some alternative approaches to measuring education and age effects.

In Table 3, I recode the two variables of contacting into a single variable that considers offline (only) contacting as the reference point and online (only) contacting as the comparison in a multinomial logistic regression model (the full model is available in Appendix E). This analysis offers another line of evidence about whether participatory inequality is distinctive in the online versus offline realm. I find that education and income differences are identical in online and offline environments. Females are more likely to contact online only than offline only. The slope for age differs for online only contacting compared to offline only contacting.

As mentioned, the theory of digitization of citizen–government contact suggests online contacting is easier, less resource-intense, and requires a lower level of motivation (RQ1). However, examining differential effects (Table 3) does not support this idea. Political interest and connecting to social groups on social media do not have differential relationships in the online and offline environment. Political efficacy has a more positive relationship with respect to online only versus offline only contacting (*p* = .054). Online news has a weaker relationship with online contacting only compared to offline only contacting (Table 3).

## Discussion and Conclusion

This paper offers a comprehensive account of participatory inequality in relation to online and offline forms of contacting officials in four countries over two time periods. I examine the differential role of demographic differences in online and offline environments, while accounting for key moderators of political interest, efficacy, information, and group ties as well as life cycle events (retirement, attending school, unemployment). I review more than 20 studies that suggest education and age differences are less likely to be significant in the online vs. offline environment, but no differences by modality in relation to income and gender. This contemporary survey research suggests: (1) socioeconomic differences are consistent in online and offline environments; (2) gender differences are substantially smaller in the online versus offline environment (except in Canada), but males are more likely to contact officials compared to females; and (3) age differences are smaller in the online versus offline environment. These findings are important as they imply the digitization of communication has reduced some of the participatory inequalities observed related to gender and age. These findings offer support for theories about the Internet offering easier access to public officials by reducing the resources, skills, and motivation needed to participate. Using cross-national survey data was important to test this theory because the existing research left gaps about whether differences relate to specific country contexts and/or to the theory of digitization online affordances and, thus, apply across contexts. I find few country-specific differences and greater support for theories about digitization.

My study is distinctive in exploring online contact. Comparing existing literature and new survey data, I find age has a consistent role across countries (see Table 4). Indeed, the strongest evidence of counter-stratification relates to age. This finding is consistent with Schlozman et al.’s (2010, 2018) findings regarding online activities and age in the United States; they argue the only evidence of counter-stratification relates to age. I extend that finding further by isolating the effects of age in a multivariate model and demonstrating the importance of age in a cross-national context, which also addresses a gap in Dalton’s (2017) analysis.

**Table 4.** Our Findings About Online Contacting Versus Existing Research.

	Other Studies (see Table 1)	USA	UK	France	Canada
Education	6 of 9 studies find significant effects	Positive, significant	*Not significant*	*Not significant*	Positive, significant
Income	4 of 8 studies find significant effects	*Not significant*	Negative, significant	*Not significant*	Negative, significant
Age	6 of 10 studies find significant effects	Negative, significant	Negative, significant	Negative, significant	Negative, significant
Females	4 of 10 studies find significant effects	Negative, significant	*Not significant*	Negative, significant	Negative, significant

Comparing Table 1 and Appendix Table A.

I also find older people are less likely to contact officials offline. However, the offline contacting and age differences may be an outcome of the reliance on an online panel. The sample may under-represent older people who engage in offline contacting and do not use the Internet (and thus are not in the online panel). I do not see this age pattern in other studies, particularly those that do not rely on online panels (e.g., Pew data, see Table 2). The objective was to examine differential effects of age in online versus offline modalities, so the sample selection worked against finding significant effects of age (because the sample is restricted to online users). Despite the sample limitation, I find substantial age effects on online contacting; these effects are much stronger than in offline contacting even among online users.

For the most part, the scholarship has sidelined questions about participatory inequality as it relates to demographics. As noted earlier, Verba et al. (1995) explain citizens' lack of participation in terms of “can’t,” “don’t want to, and “nobody asked” (p. 16), rather than demographic variables. The theory of digitization of citizen-officials contact mimics this framework. The Internet is believed to: (1) lower the effort required to participate (don’t want to); (2) offer opportunities to contact public officials (can’t); and (3) provide another forum for civic and political groups to recruit citizens to participate (nobody asked) (Best et al., 2008; Bimber, 1999; Gibson et al., 2005; Park & Perry, 2008; Schlozman et al., 2010, 2018). Even after accounting for these important factors, demographic variables persist in marking who participates and who does not. These other variables are indeed strong predictors, but are not effective in fully explaining who contacts officials online as opposed to offline. This study offers support for continued attention to age and gender in understanding patterns of participatory inequality. Furthermore, I illustrate how some of the discrepancies in the literature on online contacting relate to country-specific differences (Table 4).

In terms of study limitations, I draw upon an international scholarship when reviewing the literature and stating the hypotheses. For example, Boulding and Holzner (2020; also see Carreras, 2018) use the Latin American Public Opinion Project (LAPOP). Huang et al. (2017) study 13 countries using the Asian Barometer Survey. Lockwood and Krönke (2021) study 36 countries using the Afrobarometer. Several studies use the European Social Survey (Carreras, 2018; Grasso, 2016; Mattila & Papageorgiou, 2017; Stockemer & Rapp, 2019). Dalton (2017) and Carreras (2018) uses ISSP data, which includes 33 countries. While these are incredible data sources to document patterns of contacting, these large surveys do not have measures of activities with online equivalents and thus offer little insight into the process of digitization and its impact on participatory inequalities. The study is limited in terms of the number of countries compared to these other studies. I focus exclusively on Western democracies, yet the patterns of participatory inequality may differ across the globe. For example, Huang et al. (2017) and Lockwood and Krönke (2021) do not find income differences in contacting. The studies using the LAPOP do not show gender differences (see Table 1). In Latin America, Asia, and Africa, other factors are perhaps more important than age, gender, income, and education in marking who contacts officials and thus have influence on government policies and access to government services. Furthermore, the exclusive focus on Western democracies means we do not know the state of digital inequality in less democratic states. Political efficacy and demographic covariates may be differentially important in these political systems.

Future research should explore perceptions about the efficacy of contacting online compared to offline. The perceived effectiveness of this modality may differ when citizens are surveyed versus when officials are surveyed (Chen et al., 2018). Again, I draw on international scholarship that suggests gender differences (but not age differences) in the perceived efficacy of online forms of participation (Demertzis et al., 2013; Lee & Huang, 2014; Martin et al., 2018). This line of research could help further explain who contacts online as opposed to offline in Western democracies.

Should online and offline modes be distinguished? Dalton (2017) repeatedly refers to online contacting of officials as a continuation of offline activities into an online mode (p. 128) and Schlozman et al. (2010, 2018) call it a “repackaging” of activities into a different form. These descriptions do not fit a contemporary perspective of contacting officials. People can contact officials through social media, and this type of contact does not require the same levels of skills and resources needed to write an email or a letter. On Twitter, contact is limited to 280 characters, which hardly enables the communication of a reasoned request (or influence) related to policy. Online contact through Twitter may reduce the level of motivation required to contact officials as well as the skills required to write or reason. However, these Twitter-mediated contacts may have less influence on government policy (Chen et al., 2018). Research suggests the public opinion offered to politicians via Twitter is skewed toward the usual suspects, rather than new voices (Spierings et al., 2018). Further research should disaggregate online forms of contacting; email is not the same as Twitter posts, but Twitter communication is given a good deal of attention in the scholarship on citizen-elite communication (Jungherr, 2016). I conclude that online and offline contacting should not be aggregated into a single measure of hybrid contacting, as this would hide significant participatory differences and the differential effects of key variables (e.g., online news consumption was a stronger correlate of offline contacting than online contacting). Instead, further research should untangle the various methods of online contacting and tackle questions about the efficacy of these different online options.

## Appendix Table A

### Odds Ratio by Country

**Table:**

	Online contacting				Offline contacting			
	b	SE	ExpB	p	b	SE	ExpB	p
Education	0.178	0.046	1.194	<.001	0.152	0.049	1.164	.002
Income quint	−0.068	0.038	0.934	.072	−0.031	0.040	0.969	.440
Age6gr	−0.195	0.039	0.823	<.001	−0.266	0.042	0.767	<.001
Females1	−0.207	0.092	0.813	.025	−0.361	0.098	0.697	<.001
Student1	−0.405	0.244	0.667	.096	−0.594	0.255	0.552	.020
Unemployed1	−0.438	0.151	0.645	.004	−0.525	0.163	0.591	.001
Retired1	0.254	0.146	1.289	.082	0.182	0.159	1.199	.254
Political interest	0.476	0.057	1.610	<.001	0.384	0.062	1.468	<.001
Political efficacy	0.572	0.058	1.772	<.001	0.639	0.063	1.894	<.001
Online news	0.188	0.028	1.206	<.001	0.219	0.028	1.245	<.001
Civic groups on SM	0.509	0.095	1.664	<.001	0.553	0.100	1.739	<.001
Year 2021	0.073	0.045	1.076	.108	0.081	0.048	1.085	.093
**Above: US, *n*** = **2817**		**r** ^**2**^	**=**	**.223**		**r** ^**2**^	**=**	**.232**
Education	0.030	0.050	1.031	.544	0.075	0.056	1.078	.177
Income quint	−0.103	0.039	0.902	.009	−0.123	0.044	0.884	.005
Age6gr	−0.193	0.043	0.824	<.001	−0.372	0.049	0.689	<.001
Females1	−0.132	0.105	0.876	.208	−0.292	0.119	0.746	.014
Student1	−0.439	0.265	0.645	.098	−0.961	0.296	0.383	.001
Unemployed1	−0.433	0.185	0.648	.019	−0.651	0.210	0.522	.002
Retired1	0.358	0.160	1.430	.025	0.434	0.188	1.543	.021
Political interest	0.373	0.066	1.453	<.001	0.383	0.075	1.467	<.001
Political efficacy	0.535	0.068	1.708	<.001	0.587	0.076	1.799	<.001
Online news	0.305	0.036	1.357	<.001	0.332	0.038	1.393	<.001
Civic groups on SM	0.704	0.102	2.021	<.001	0.599	0.114	1.821	<.001
Year 2021	−0.051	0.049	0.950	.301	−0.091	0.055	0.913	.102
**Above: UK, *n*** = **2496**		**r** ^**2**^	**=**	**.187**		**r** ^**2**^	**=**	**.200**
Education	−0.016	0.050	0.984	.749	−0.070	0.054	0.932	.192
Income quint	−0.049	0.043	0.952	.250	−0.067	0.046	0.935	.142
Age6gr	−0.195	0.048	0.823	<.001	−0.284	0.051	0.753	<.001
Females1	−0.469	0.102	0.625	<.001	−0.518	0.110	0.596	<.001
Student1	−0.553	0.247	0.575	.025	−0.567	0.252	0.567	.024
Unemployed1	−0.302	0.223	0.739	.177	−0.358	0.239	0.699	.134
Retired1	0.284	0.162	1.328	.080	0.259	0.178	1.296	.145
Political interest	0.300	0.059	1.349	<.001	0.309	0.064	1.363	<.001
Influence govt	0.490	0.062	1.632	<.001	0.496	0.066	1.641	<.001
Online news	0.248	0.033	1.281	<.001	0.246	0.035	1.280	<.001
Civic groups on SM	0.438	0.101	1.550	<.001	0.533	0.108	1.705	<.001
Year 2021	−0.061	0.049	0.941	.210	−0.111	0.052	0.895	.034
**Above: FR, *n*** = **2614**		**r** ^**2**^	**=**	**.155**		**r** ^**2**^	**=**	**.161**
Education	0.115	0.052	1.122	.028	0.115	0.056	1.122	.042
Income quint	−0.093	0.039	0.911	.016	−0.081	0.042	0.922	.051
Age6gr	−0.307	0.045	0.736	<.001	−0.307	0.049	0.736	<.001
Females1	−0.245	0.103	0.783	.018	−0.136	0.112	0.873	.223
Student1	−0.494	0.224	0.610	.027	−0.563	0.239	0.569	.019
Unemployed1	−0.052	0.190	0.950	.787	−0.256	0.212	0.774	.228
Retired1	0.544	0.158	1.724	.001	0.500	0.174	1.648	.004
Political interest	0.539	0.066	1.714	<.001	0.397	0.072	1.488	<.001
Political efficacy	0.435	0.067	1.546	<.001	0.441	0.073	1.554	<.001
Online news	0.149	0.031	1.161	<.001	0.237	0.032	1.268	<.001
Civic groups on SM	0.721	0.102	2.056	<.001	0.692	0.112	1.997	<.001
Year 2021	0.065	0.049	1.067	.189	0.068	0.054	1.070	.204
**Above: CA, *n*** = **2626**		**r** ^**2**^	**=**	**.163**		**r** ^**2**^	**=**	**.144**

SM, social media; US, United States; UK, United Kingdom; FR, France; CA, Canada. Cox & Snell r-square.

## Appendix B

### Comparison of Online and Offline Contact Model

**Table:**

	Online contacting				Offline contacting			
	b	SE	ExpB	p	b	SE	ExpB	p
Education	0.085	0.024	1.089	<.001	0.077	0.026	1.080	.003
Income quint	−0.074	0.019	0.928	<.001	−0.068	0.021	0.934	.001
Age6gr	−0.218	0.021	0.804	<.001	−0.303	0.023	0.739	<.001
Females1	−0.246	0.049	0.782	<.001	−0.308	0.054	0.735	<.001
Student1	−0.469	0.120	0.626	<.001	−0.647	0.128	0.523	<.001
Unemployed1	−0.322	0.090	0.725	<.001	−0.457	0.099	0.633	<.001
Retired1	0.361	0.077	1.435	<.001	0.350	0.086	1.418	<.001
United States	0.307	0.066	1.360	<.001	0.339	0.072	1.403	<.001
United Kingdom	0.320	0.069	1.377	<.001	0.244	0.076	1.276	.001
France	0.314	0.070	1.369	<.001	0.422	0.076	1.525	<.001
Political interest	0.416	0.031	1.515	<.001	0.360	0.034	1.434	<.001
Political efficacy	0.517	0.031	1.677	<.001	0.550	0.034	1.733	<.001
Online news	0.213	0.016	1.237	<.001	0.251	0.016	1.285	<.001
Civic groups on SM	0.589	0.050	1.802	<.001	0.592	0.054	1.808	<.001
Year 2021	0.011	0.024	1.011	.654	−0.009	0.026	0.991	.740
***n*** = **10,553**		**r** ^**2**^	**=**	**.183**		**r** ^**2**^	**=**	**.186**

SM, social media. Cox & Snell r-square. Canada is the reference group for the above analysis.

## Appendix C

### Comparison of Online and Offline Contact Model Treating Education and Age as Non-Linear

**Table:**

	Online contacting				Offline contacting			
	b	SE	ExpB	p	b	SE	ExpB	p
Some college	0.115	0.068	1.122	.090	0.089	0.074	1.093	.228
Bachelor’s degree	0.101	0.062	1.107	.103	0.092	0.067	1.096	.174
More than a bachelor’s degree	0.370	0.085	1.448	<.001	0.339	0.091	1.403	<.001
Income quint	−0.068	0.019	0.934	<.001	−0.062	0.021	0.940	.003
Age6gr	−0.283	0.044	0.753	<.001	−0.303	0.048	0.739	<.001
Age-squared	0.055	0.009	1.056	<.001	0.057	0.010	1.059	<.001
Females1	−0.256	0.050	0.774	<.001	−0.318	0.054	0.728	<.001
Student1	−0.711	0.128	0.491	<.001	−0.884	0.136	0.413	<.001
Unemployed1	−0.330	0.091	0.719	<.001	−0.469	0.100	0.626	<.001
Retired1	0.153	0.088	1.166	.082	0.118	0.099	1.125	.233
United States	0.298	0.067	1.347	<.001	0.332	0.073	1.394	<.001
United Kingdom	0.326	0.070	1.385	<.001	0.249	0.077	1.283	.001
France	0.313	0.071	1.368	<.001	0.420	0.077	1.522	<.001
Political interest	0.409	0.031	1.505	<.001	0.353	0.034	1.423	<.001
Political efficacy	0.515	0.032	1.674	<.001	0.548	0.034	1.730	<.001
Online news	0.218	0.016	1.243	<.001	0.256	0.016	1.292	<.001
Civic groups on SM	0.585	0.050	1.794	<.001	0.587	0.054	1.799	<.001
Year 2021	0.009	0.024	1.009	.715	−0.011	0.026	0.989	.672
***n* = 10,553**		**r** ^**2**^	**=**	**.186**		**r** ^**2**^	**=**	**.189**

Cox & Snell r-square. SM, social media. Reference groups for the above: high school or less; males; non-students; Canada. Age-squared is calculated as age×ln(age).

## Appendix D

### Comparison of Online and Offline Contact Model Treating Education and Age as Categorical

**Table:**

	Online contacting				Offline contacting			
	b	SE	ExpB	p	b	SE	ExpB	p
Some college	0.102	0.068	1.108	.130	0.072	0.074	1.075	.328
Bachelor’s degree	0.089	0.062	1.093	.151	0.078	0.067	1.081	.248
More than a bachelor’s degree	0.361	0.085	1.435	<.001	0.326	0.091	1.385	<.001
Income quint	−0.070	0.019	0.932	<.001	−0.067	0.021	0.936	.002
Ages 18 to 24	0.970	0.127	2.637	<.001	1.344	0.137	3.835	<.001
Ages 25 to 34	0.558	0.112	1.747	<.001	0.760	0.124	2.139	<.001
Ages 35 to 44	0.271	0.113	1.311	.016	0.566	0.125	1.762	<.001
Ages 45 to 54	−0.042	0.111	0.958	.703	0.051	0.125	1.052	.683
Ages 55 to 64	−0.027	0.092	0.973	.767	−0.076	0.106	0.927	.474
Females1	−0.260	0.050	0.771	<.001	−0.317	0.054	0.728	<.001
Student1	−0.624	0.129	0.536	<.001	−0.813	0.136	0.444	<.001
Unemployed1	−0.330	0.091	0.719	<.001	−0.470	0.100	0.625	<.001
Retired1	0.104	0.092	1.110	.259	0.065	0.104	1.067	.536
United States	0.295	0.067	1.344	<.001	0.323	0.073	1.382	<.001
United Kingdom	0.315	0.070	1.370	<.001	0.234	0.077	1.264	.002
France	0.303	0.071	1.353	<.001	0.410	0.077	1.507	<.001
Political interest	0.407	0.031	1.503	<.001	0.354	0.034	1.424	<.001
Political efficacy	0.515	0.032	1.674	<.001	0.548	0.034	1.730	<.001
Online news	0.217	0.016	1.242	<.001	0.253	0.016	1.288	<.001
Civic groups on SM	0.591	0.050	1.806	<.001	0.596	0.054	1.815	<.001
Year 2021	0.009	0.024	1.009	.701	−0.011	0.026	0.989	.678
***n* = 10,553**		**r** ^**2**^	**=**	**.186**		**r** ^**2**^	**=**	**.189**

Cox & Snell r-square. SM, social media. Reference groups for the above: high school or less; males; non-students; Canada; and seniors aged 65 years or more.

## Appendix E

### Multinomial Logistic Regression with Offline Contact as the Reference

**Table:**

	Online only (8.1%)				No modes (68.3%)				Both modes (21.6%)			
	b	SE	ExpB	p	b	SE	ExpB	p	b	SE	ExpB	p
Education	0.033	0.079	1.034	.673	−0.036	0.072	0.964	.617	0.057	0.074	1.059	.442
Income quint	0.036	0.063	1.037	.569	0.116	0.058	1.123	.045	0.039	0.059	1.040	.512
Age6gr	0.279	0.069	1.322	<.001	0.339	0.062	1.404	<.001	0.032	0.064	1.032	.621
Females1	0.416	0.163	1.515	.011	0.609	0.149	1.839	<.001	0.304	0.153	1.355	.047
Student1	1.013	0.470	2.753	.031	1.231	0.436	3.426	.005	0.598	0.443	1.819	.177
Unemployed1	0.308	0.277	1.361	.266	0.330	0.252	1.391	.190	−0.147	0.262	0.863	.573
Retired1	−0.207	0.254	0.813	.414	−0.556	0.234	0.574	.018	−0.173	0.242	0.841	.475
United States	−0.131	0.217	0.877	.546	−0.318	0.198	0.728	.108	0.055	0.203	1.056	.788
United Kingdom	0.243	0.232	1.275	.294	−0.073	0.214	0.930	.733	0.242	0.221	1.274	.272
France	−0.243	0.227	0.784	.284	−0.293	0.204	0.746	.152	0.156	0.211	1.169	.461
Political interest	0.150	0.097	1.162	.121	−0.269	0.088	0.764	.002	0.164	0.091	1.178	.072
Influence govt	0.191	0.099	1.211	.054	−0.041	0.090	0.960	.649	0.605	0.093	1.832	<.001
Online news	−0.109	0.048	0.897	.024	−0.198	0.043	0.821	<.001	0.074	0.044	1.077	.088
Civic groups on SM	−0.101	0.162	0.904	.533	−0.597	0.147	0.551	<.001	0.074	0.152	1.077	.626
Year 2021	−0.028	0.077	0.973	.722	−0.097	0.071	0.908	.171	−0.105	0.073	0.900	.147

Reference is offline contact only (2.0% of sample). SM, social media. For countries, Canada is the reference group. Cox & Snell r-square = 0.219, *n* = 10,553.

## References

[bibr1-08944393211071067] Aars J. Stromsnes K. (2007). Contacting as a channel of political involvement: Collectively motivated, individually enacted. West European Politics, 30(1), 93–120. 10.1080/01402380601019704

[bibr2-08944393211071067] Anduiza E. Gallego A. Cantijoch M. (2010). Online political participation in Spain: The impact of traditional and Internet resources, Journal of Information Technology & Politics, 7(4), 356–368. 10.1080/19331681003791891

[bibr3-08944393211071067] Beam M. A. Child J. T. Hutchens M. J. Hmielowski J. D. (2018). Context collapse and privacy management: Diversity in Facebook friends increases online news reading and sharing. New Media & Society, 20(7), 2296–2314. 10.1177/1461444817714790

[bibr4-08944393211071067] Best S. J. Krueger B. S. Ladewig J. (2008) The effect of risk perceptions on online political participatory decisions. Journal of Information Technology & Politics, 4(1), 5–17. 10.1300/J516v04n01_02

[bibr5-08944393211071067] Bimber B. (1999). The Internet and citizen communication with government: Does the medium matter? Political Communication, 16(4), 409–428. 10.1080/105846099198569

[bibr6-08944393211071067] Boulding C. Holzner C. (2020). Community organizations and Latin America’s poorest citizens: voting, protesting, and contacting government. Latin American Politics and Society, 62(4), 98–125. 10.1017/lap.2020.17

[bibr7-08944393211071067] Boulianne S. (2011). Stimulating or reinforcing political interest: Using panel data to examine the use of news media and political interest. Political Communication, 28(2), 147–162. 10.1080/10584609.2010.540305

[bibr8-08944393211071067] Boulianne S. (2020). Twenty years of digital media effects on civic and political participation. Communication Research, 47(7), 947–966. 10.1177/0093650218808186

[bibr9-08944393211071067] Boulianne S. Theocharis Y. (2020). Young people, digital media and engagement: A meta-analysis of research. Social Science Computer Review, 38(2), 111–127. 10.1177/0894439318814190

[bibr10-08944393211071067] Brundidge J. Baek K. Johnson T. J. Williams L. (2013). Does the medium still matter? The influence of gender and political connectedness on contacting U.S. public officials online and offline. Sex Roles, 69(1-2), 3–15. 10.1007/s11199-013-0280-5

[bibr11-08944393211071067] Buchi M. Vogler F. (2017). Testing a digital inequality model for online political participation. Socius, 3(3), 1–13. 10.1177/2378023117733903

[bibr12-08944393211071067] Cao X. X. Brewer P. R. (2008). Political comedy shows and public participation in politics. International Journal of Public Opinion Research, 20(1), 90–99. 10.1093/ijpor/edm030

[bibr13-08944393211071067] Carreras M. (2018). Why no gender gap in electoral participation? A civic duty explanation. Electoral Studies, 52(April), 36–45. 10.1016/j.electstud.2018.01.007.

[bibr14-08944393211071067] Chen K. Lee N. Marble W. (2018). Do elected officials listen to constituents on social media? Survey evidence from local politicians in the United States. Available at SSRN: https://ssrn.com/abstract=3251651

[bibr15-08944393211071067] Dalton R. J. (2017). The participation gap: Social status and political inequality. : Oxford University Press.

[bibr16-08944393211071067] Demertzis N. Milioni D. L. Gialamas V. (2013). Internet use and political efficacy: The case of Cyprus. International Journal of Electronic Governance, 6(3), 187–208. 10.1504/IJEG.2013.058413

[bibr17-08944393211071067] Filetti A. (2016). Participating unequally? Assessing the macro-micro relationship between income inequality and political engagement in Europe. PACO, 9(1), 72–100. 10.1285/i20356609v9i1p72

[bibr18-08944393211071067] Fishkin J. S. (2009). When the people speak: Deliberative democracy and public consultation. Oxford University Press.

[bibr19-08944393211071067] Franzen A. Meyer R. (2010). Environmental attitudes in cross-national perspective: A multilevel analysis of the ISSP 1993 and 2000. European Sociological Review, 26(2), 219–234. 10.1093/esr/jcp018

[bibr20-08944393211071067] Gastil J. Knobloch K. Kelly M. (2012). Evaluating deliberative public events and projects. In Nabatchi T. Gastil J. Weiksner G. M. Leighninger M. (Eds.), Democracy in motion: Evaluating the practice and impact of deliberative civic engagement (pp. 205–230). Oxford University Press.

[bibr21-08944393211071067] Gibson R. K. Lusoli W. Ward S. (2005). Online participation in the UK: Testing a ‘contextualised’ model of Internet effects. British Journal of Politics & International Relations, 7(4), 561–583. 10.1111/j.1467-856x.2005.00209.x

[bibr22-08944393211071067] Grasso M. (2016). Generations, political participation and social change in Western Europe*.* Routledge.

[bibr23-08944393211071067] Ha S. Kim S. Jo S. (2013). Personality traits and political participation: Evidence from South Korea. Political Psychology, 34(4), 511–532. 10.1111/pops.12008

[bibr24-08944393211071067] Hargittai E. (2002). Second-level digital divide: Differences in people’s online skills. First Monday, 7(4). https://doi.org/10.5210%2Ffm.v7i4.942

[bibr25-08944393211071067] Hargittai E. (2010). Digital na(t)ives? Variation in Internet skills and uses among members of the ‘Net Generation. Sociological Inquiry, 80(1), 92–113. 10.1111/j.1475-682X.2009.00317.x

[bibr26-08944393211071067] Heger K. Hoffmann C. P. (2019). Feminism! What is it good for? The role of feminism and political self-efficacy in women’s online political participation. Social Science Computer Review. 10.1177/0894439319865909

[bibr27-08944393211071067] Holt K. Shehata A. Strömbäck J. Ljungberg E. (2013). Age and the effects of news media attention and social media use on political interest and participation: Do social media function as leveller? European Journal of Communication, 28(1), 19–34. 10.1177/0267323112465369

[bibr28-08944393211071067] Hsieh Y. P. Li M.H. (2014). Online political participation, civic talk, and media multiplexity: How Taiwanese citizens express political opinions on the Web. Information, Communication & Society, 17(1), 26–44. 10.1080/1369118X.2013.833278

[bibr29-08944393211071067] Huang M. H. Whang T. Lei X. C. (2017). The Internet, social capital, and civic engagement in Asia. Social Indicators Research, 132(2), 559–578. 10.1007/s11205-016-1319-0

[bibr30-08944393211071067] Jungherr A. (2016). Twitter use in election campaigns: A systematic literature review. Journal of Information Technology & Politics, 13(1), 72–91. 10.1080/19331681.2015.1132401

[bibr31-08944393211071067] Lee C. Huang T. (2014). E-government use and citizen empowerment: Examining the effects of online information on political efficacy. Electronic Journal of E-Government, 12(1), 54. http://www.ejeg.com/main.html

[bibr32-08944393211071067] Leighley J. E. Oser J. (2018). Representation in an era of political and economic inequality: How and when citizen engagement matters. Perspectives on Politics, 16(2), 328–344. 10.1017/s1537592717003073

[bibr33-08944393211071067] Li Y. Marsh D. (2008). New forms of political participation: Searching for expert citizens and everyday makers. British Journal of Political Science, 38(2), 247–272. 10.1017/S0007123408000136

[bibr34-08944393211071067] Lockwood S. Krönke M. (2021). Do electoral systems affect how citizens hold their government accountable? Evidence from Africa. Democratization, 28(3), 583–603. 10.1080/13510347.2020.1840556

[bibr35-08944393211071067] Martin J. D. Martins R. J. Naqvi S. (2018). Media use predictors of online political efficacy among Internet users in five Arab countries. Information, Communication & Society, 21(1), 129–146. 10.1080/1369118X.2016.1266375

[bibr36-08944393211071067] Mattes R. (2015). South Africa’s emerging black middle class: A harbinger of political change? Journal of International Development, 27(5), 665–692. 10.1002/jid.3100

[bibr37-08944393211071067] Mattila M. Papageorgiou A. (2017). Disability, perceived discrimination and political participation. International Political Science Review, 38(5), 505–519. 10.1177/0192512116655813

[bibr38-08944393211071067] Mossberger K. Tolbert C. J. McNeal R. S. (2008). Digital citizenship: The Internet, society, and participation. Massachusetts Institute of Technology.

[bibr39-08944393211071067] National Institute of Statistics and Economic Studies . (2018). Demographic Balance Sheet. https://www.insee.fr/en/statistiques/2382609?sommaire=2382613

[bibr40-08944393211071067] Nie N. H. Junn J. Stehlik-Barry K. (1996). Education and democratic citizenship in America. University of Chicago Press.

[bibr41-08944393211071067] Office of National Statistics . (2016). Population estimates for the UK, England and Wales, Scotland and Northern Ireland: mid-2016. https://www.ons.gov.uk/peoplepopulationandcommunity/populationandmigration/populationestimates/bulletins/annualmidyearpopulationestimates/mid2016#main-points

[bibr42-08944393211071067] Olcese C. Saunders C. Tzavidis N. (2014). In the streets with a degree: How political generations, educational attainment and student status affect engagement in protest politics. International Sociology, 29(6), 525–545. 10.1177/0268580914551305

[bibr43-08944393211071067] Oser J. Leighley J. E. Winneg K. M. (2014). Participation, online and otherwise: What’s the difference for policy preferences? Social Science Quarterly, 95(5), 1259–1277. 10.1111/ssqu.12100

[bibr44-08944393211071067] Park H. M. Perry J. L. (2008). Does Internet use really facilitate civic engagement? Empirical evidence from the American National Election Studies. In Yang K. Bergrud E. (Eds.), Civic engagement in a network society (pp. 237–269). Information Age Publishing.

[bibr45-08944393211071067] Putnam R. D. (1993). Making democracy work: Civic traditions in modern Italy. Princeton University Press.

[bibr46-08944393211071067] Putnam R. D. (2000). Bowling alone: The collapse and revival of American community. Touchstone.

[bibr47-08944393211071067] Rueß C. Heger K. Hoffmann C. Boulianne S. (2019). Online political participation – The Evolution of a concept*.* paper presented at the International Communication Association annual meeting.

[bibr48-08944393211071067] Schlozman K. L. Brady H. Verba S. (2018). Unequal and unrepresented: Political inequality and the people’s voice in the new gilded age. Princeton University Press.

[bibr49-08944393211071067] Schlozman K. L. Verba S. Brady H. (2010). Weapon of the strong? Participatory inequality and the Internet. Perspectives on Politics, 8(2), 487–509. 10.1017/S1537592710001210

[bibr50-08944393211071067] Smets K. van Ham C. (2013). The embarrassment of riches? A meta-analysis of individual-level research on voter turnout. Electoral Studies, 32(2), 344–359. 10.1016/j.electstud.2012.12.006

[bibr51-08944393211071067] Spierings N. Jacobs K. Linders N. (2018). Keeping an eye on the people: Who has access to MPs on Twitter? Social Science Computer Review, 37(2): 160–177. 10.1177/0894439318763580

[bibr52-08944393211071067] Statistics Canada . (2016). 2016 Census of Population – age and sex. https://www12.statcan.gc.ca/datasets/Index-eng.cfm?Temporal=2016&Theme=115&VNAMEE=&GA=-1&S=0

[bibr53-08944393211071067] Stockemer D. Rapp C. (2019). The influence of various measures of health on different types of political participation. Politics, 39(4) 480–513. 10.1177/0263395719844700

[bibr54-08944393211071067] Sylvester D. E. McGlynn A. J. (2010). The digital divide, political participation, and place. Social Science Computer Review, 28(1), 64–74. 10.1177/0894439309335148

[bibr55-08944393211071067] Thananithichot S. (2012). Political engagement and participation of Thai citizens: The rural–urban disparity, Contemporary Politics, 18(1), 87–108. 10.1080/13569775.2012.651274

[bibr56-08944393211071067] US Census . (2019). American community survey: Demographic and housing estimates https://data.census.gov/cedsci/table?d=ACS1-YearEstimatesDataProfiles&tid=ACSDP1Y2019.DP05&hidePreview=true

[bibr57-08944393211071067] Vaccari C. Valeriani A. Barbera P. Bonneau R. Jost J. T. Nagler J. Tucker J. A. (2015). Political expression and action on social media: Exploring the relationship between lower- and higher-threshold political activities among Twitter users in Italy. Journal of Computer-Mediated Communication, 20(1), 221–239. 10.1111/jcc4.12108

[bibr58-08944393211071067] Verba S. Burns N. Schlozman K. L. (1997). Knowing and caring about politics: Gender and political engagement. Journal of Politics, 59(4), 1051–1072. 10.2307/2998592

[bibr59-08944393211071067] Verba S. Schlozman K. L. Brady H. (1995). Voice and equality: Civic voluntarism in American politics. Harvard University Press.

[bibr60-08944393211071067] Youniss J. (2009). Why we need to learn more about youth civic engagement. Social Forces, 88(2), 971–976. 10.1353/sof.0.0253

